# Differentiation timing-dependent axon targeting and subtype specification in retinal ganglion cells

**DOI:** 10.3389/fnins.2026.1733811

**Published:** 2026-02-10

**Authors:** Lena Iwai, Tatsumi Hirata

**Affiliations:** 1 Brain Function Laboratory, National Institute of Genetics, Mishima, Shizuoka, Japan; 2 The Graduate University for Advanced Studies, SOKENDAI, Hayama, Kanagawa, Japan

**Keywords:** CreER, lateral geniculate nucleus, medial terminal nucleus, Neurod1, neurogenic tagging mouse, retinal ganglion cell, subtype specification, visual system

## Abstract

Subtypes of retinal ganglion cells (RGCs) in the mouse retina are each tuned to particular visual features and contribute to parallel visual processing in the brain. We addressed how RGCs are specified into distinct and diverse subtypes based on their differentiation timing. We used a neurogenic tagging mouse line, Neurod1^CreER^ (D1B), in which tamoxifen-inducible CreER was driven by a putative Neurod1 enhancer. Timed tamoxifen injection in this mouse line induced CreER-loxP recombination in neurons that shared the same differentiation timing. This analysis revealed that RGC axon projections to the lateral geniculate nucleus and medial terminal nucleus were segregated depending on the stage of tamoxifen injection. We further characterized the properties of these neurogenically tagged RGCs based on their molecular markers and morphological features. Our study extends the concept that differentiation timing is linked to the specification of RGC subtypes.

## 1 Introduction

The vertebrate retina is composed of diverse neuronal classes. In the mouse retina, there are five major classes of neurons: photoreceptors, horizontal cells, bipolar cells, amacrine cells, and retinal ganglion cells (RGCs) (Zeng and Sanes, 2017). RGCs represent a highly heterogeneous population with extensive functional diversity in visual information processing. Different RGC subtypes are sensitive to distinct visual features and thus transmit feature-extracted images of the world via their axons, which travel to visual centers. Each RGC subtype has historically been characterized by its morphology, gene expression, physiological properties, and axon projection patterns in the brain (Sanes and Masland, 2015). Recently, RNA-seq–based transcriptomic analyses have revealed a much finer subdivision into distinct molecular subtypes (Baden et al., 2016; Rheaume et al., 2018; Tran et al., 2019; Goetz et al., 2022). Current classifications suggest the existence of approximately 30–46 distinct RGC subtypes.

An unexplored issue is how RGC subtypes acquire distinct and diverse identities. RGCs are generated from retinal progenitors over a protracted period, from embryonic day (E)12 to E17 in mice (Young, 1985). From a neurodevelopmental perspective, the chronological timing of differentiation is a key determinant of neuronal subtype specification. For example, in the cortex, neurogenic timing determines the layer positioning, connection patterns, and molecular and physiological properties of neurons (McConnell, 1989; Lodato and Arlotta, 2015). Thus, such a mechanism may represent a conserved strategy across various nervous systems (Suzuki and Hirata, 2013; Hirata et al., 2019), we investigated whether the differentiation timing of RGCs is linked to subtype specification.

For this purpose, we used a genetically modified “neurogenic tagging” mouse line that labels neuronal subtypes according to the timing of neuronal differentiation (Hirata et al., 2019, 2021). The design of this system was based on tamoxifen (TM)-inducible CreER recombination. CreER is expressed only transiently within a short time window immediately after neuronal fate commitment. A single administration of TM at a defined developmental stage induces irreversible recombination of loxP sequences only in neurons that express CreER at the time of TM administration. Among several “neurogenic tagging” mouse lines, we selected the Neurod1^CreER^ (D1B) line, in which CreER is driven by a putative enhancer of the Neurod1 gene because it most effectively labels RGCs. The Neurod1 gene is a basic-helix–loop–helix transcription factor that is transiently expressed in neurons, including those in the retina, during the maturation phase (Morrow et al., 1998; Kiyama et al., 2011; Aprea et al., 2014). Single-cell RNA sequencing studies of the retina have shown that Neurod1 expression is dynamically regulated across developmental stages and cell types (Clark et al., 2019; Chen et al., 2021; Wu et al., 2021). During embryonic stages, Neurod1 expression is enriched in intermediate transitional retinal precursor cells, including populations that give rise to RGCs (Clark et al., 2019; Wu et al., 2021). It is detectable in embryonic stages and increases substantially during postnatal stages in clusters prominently associated with photoreceptor lineages (Clark et al., 2019; Tran et al., 2019; Chen et al., 2021). Using the Neurod1^CreER^ (D1B) line, we demonstrated that axonal projections of RGCs are segregated depending on their differentiation timing. Based on the molecular and morphological analyses of labeled RGCs, we discuss the potential RGC subtypes investigated in this study.

## 2 Results

### 2.1 Neurogenic tagging using the Neurod1^CreER^ (D1B) driver mouse

**Figure 1:**
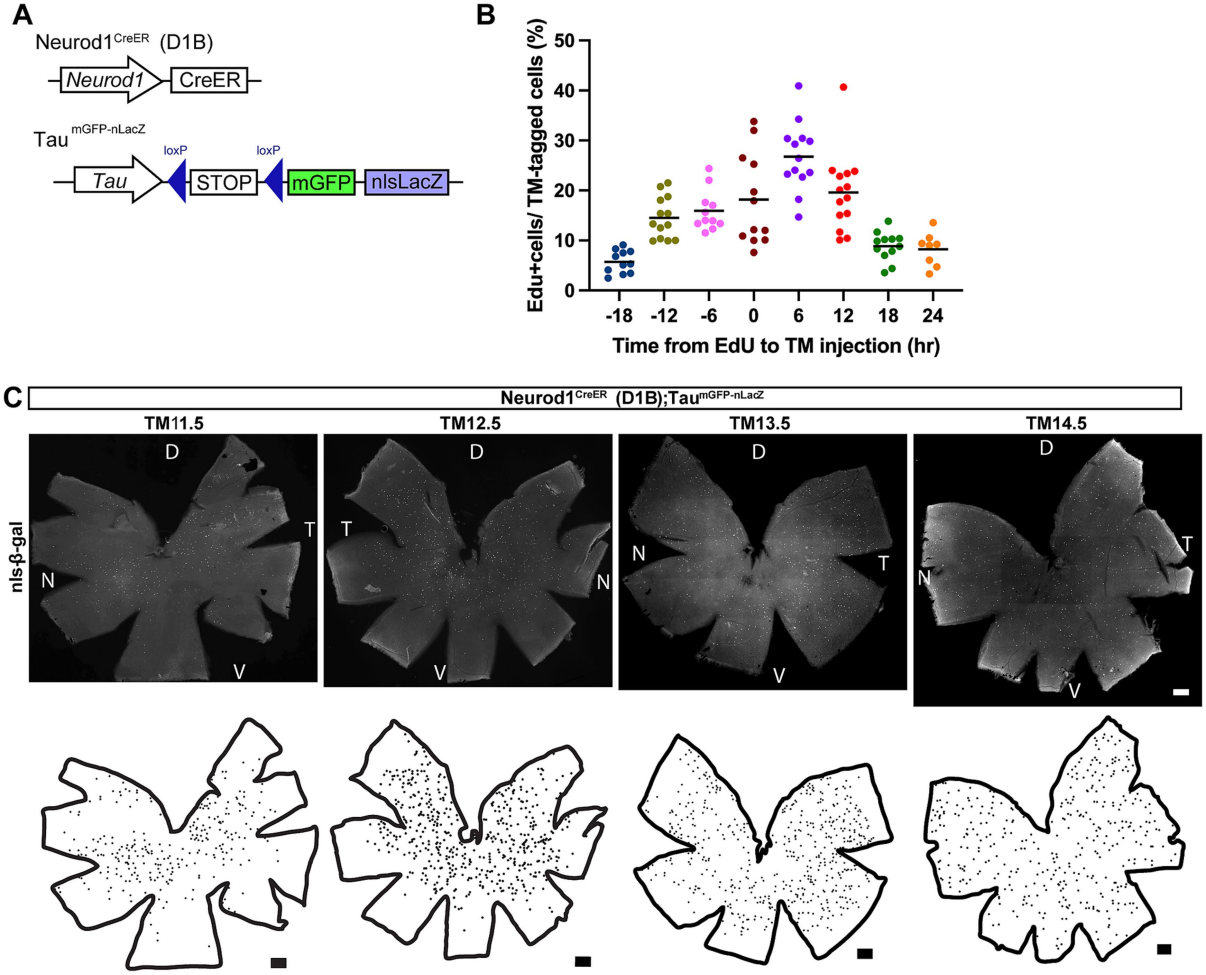
Neurogenic tagging by using the Neurod1^CreER^ (D1B) driver mouse. **(A)** Schematic gene structures of the neurogenic tagging driver Neurod1^CreER^ (D1B) and the Tau ^mGFP-nLacZ^ reporter. **(B)** Proportions of EdU- and nls-β-galactosidase double-positive neurons in the ganglion cell layer. Tamoxifen (TM) injection was administered at a fixed time point (E13.5), and EdU was administered at the indicated time point before (plus) or after (minus) TM injection. Retinal sections at P0 were immunostained to detect labeled cells. Dots indicate values from individual mice, and horizontal lines indicate means. The number of animals analyzed is as follows: *n* = 11(−18 h), 13 (−12 h), 11 (−6 h), 12 (0 h), 13 (6 h), 14 (12 h), 12 (18 h), and 8 (24 h). **(C)** Flat-mounted retinas and corresponding illustrations showing the location of nucleus-localized β-galactosidase (nls-β-gal)-positive cells at P21 (white dots in micrographs and black dots in illustrations). The TM-tagged stages are indicated at the top. The orientations of the illustrations correspond to the micrographs shown above. D, dorsal; T, temporal; N, nasal; V, ventral. Scale bars, 300 μm.

We first examined RGC labeling using four neurogenic tagging mouse lines (Hirata et al., 2021)^1^ and found that the Neurod1^CreER^ (D1B) line labeled RGCs most effectively. To obtain a fine temporal profile of labeling in the retina, we crossed the Neurod1^CreER^ (D1B) driver line with the Tau^mGFP-nLacZ^ reporter mouse, which expresses dual nucleus- and membrane-localized reporters under a constitutive neuronal promoter after excision of the loxP-STOP-loxP cassette (Hippenmeyer et al., 2005) (Figure 1A). The Tau-driven reporter has been widely used to label differentiated neurons in multiple contexts, such as neurons in the interpeduncular nucleus (Ruiz-Reig et al., 2019), the jugular and nodose ganglia (Lowenstein et al., 2024), and the spinal cord (Xu et al., 2013). In our study, when a ubiquitous Cre driver line, CAG-Cre (Sakai and Miyazaki, 1997; Sato et al., 2011), was crossed with the Tau reporter line, strong reporter expression was predominantly observed in the ganglion cell layer (GCL; Supplementary Figure 1). This indicates that Tau-driven reporter expression is slightly biased toward RGCs in the retina.

In the Neurod1^CreER^ (D1B) driver line with the Tau^mGFP-nLacZ^ reporter mouse, reporter expression driven by the Tau locus was also confined to the GCL when Cre recombination was induced between E11.5 and E15.5 (hereafter referred to as TM11.5 and TM15.5, respectively; Supplementary Figure 1). In this context, we found that more than 85% of reporter-positive cells co-expressed the established RGC marker, RBPMS (Supplementary Figure 1). A single administration of TM at E13.5 most abundantly induced loxP recombination in RGCs (approximately 440 neurons per flat-mount retina; see also Supplementary Figure 1), whereas fewer recombination events were observed at earlier and later stages. This temporal profile corresponds well with the timing of RGC production previously described in the developing mouse retina (Young, 1985).

At the time of TM administration at E13.5, we characterized the cell cycle status of labeled RGCs using the thymidine analog EdU (Figure 1B), and the labeled cells were subsequently analyzed at P0. The results indicated that RGCs were most susceptible to TM-induced recombination 6 h after the final DNA synthesis. This result also suggested that a fraction of recombination may have occurred in the progenitor stage because some of the RGCs incorporated EdU, even after TM administration. This interpretation is consistent with a previous report showing that Neurod1 is expressed predominantly in postmitotic neurons but occasionally in mitotically active cells in the developing mouse retina (Liu et al., 2008). The same conclusion is also supported by RNA-seq-based transcriptomic data (Clark et al., 2019; Chen et al., 2021; Wu et al., 2021).

We next examined the spatial distribution of the neurogenic-tagged RGCs in flat-mount retinas at P21 (Figure 1C). Previous birthdating studies have shown that RGCs are generated from the center to the periphery in a wave-like pattern (Young, 1985; Lyu and Mu, 2021). Although this trend was not very clear in our analyses, the early tagged (TM11.5) neurons were more concentrated in the central retina, whereas the later-tagged (TM14.5) neurons were distributed more widely across the retina (Figure 1C). Because RGC subtypes were not distinguished in this analysis, the overlapping distributions of individual subtypes may have reduced the spatial resolution of the individual differentiation waves.

### 2.2 Axon projections of neurogenic-tagged RGCs

**Figure 2:**
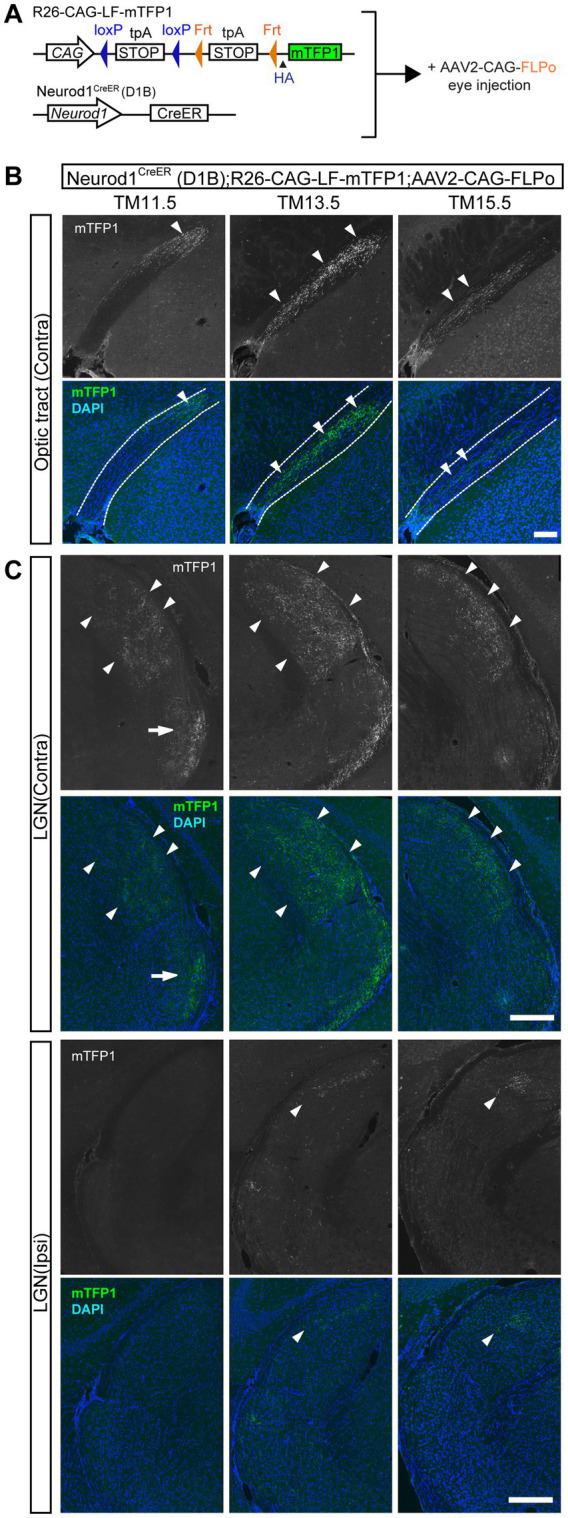
Axon projections of the neurogenic-tagged RGCs in the optic tract and LGN. **(A)** Schematic gene structures of the neurogenic tagging driver Neurod1^CreER^ (D1B) and R26-CAG-LF-mTFP1 reporter. Tamoxifen (TM) injection was administered at E11.5, E13.5, or E15.5 to induce CreER recombination. AAV2-CAG-FLPo was injected intravitreally at the postnatal stage to induce Flp recombination in an eye-specific manner. **(B,C)** Labeling of TM-tagged axon arbors by mTFP1 immunostaining (white in the upper panels, green in the lower panels) in brain sections. The TM-tagging stages are indicated at the top. DAPI staining is shown in blue. **(B)** Labeled axons in the contralateral optic tract (arrowheads) at P28 for TM11.5, P29 for TM13.5, and P32 for TM15.5. The dotted line indicates the optic tract border. **(C)** Labeled axons in the dorsal lateral geniculate nucleus (dLGN; arrowheads) and ventral LGN (vLGN; arrows) at P28 for TM11.5, P29 for TM13.5, and P32 for TM15.5. Contra, contralateral projections; Ipsi, ipsilateral projections. Scale bars, 250 μm.

RGC axons from the eye project through the optic tract to the retinorecipient brain targets (Morin and Studholme, 2014; Martersteck et al., 2017; Kerschensteiner and Feller, 2024). We examined where the neurogenic-tagged RGCs project their axons. To label axons in an eye-specific manner, Neurod1^CreER^ (D1B) mice were crossed with R26-CAG-LF-mTFP1 reporter mice, in which monomeric Teal Fluorescent Protein 1 (mTFP1; Clavularia cFP484) was expressed after the removal of double STOP cassettes by two recombinases, Cre and Flippase (Figure 2A) (Imayoshi et al., 2012). We chose to analyze three stages of TM administration, TM11.5, TM13.5, and TM15.5, as a two-day interval could reveal differences, if any. After the Cre-loxP STOP cassette was removed by TM in Neurod1^CreER^ (D1B) mice, an AAV2 vector coding Flp recombinase (AAV2-CAG-FLPo) was intravitreally injected into one eye at the postnatal stage, and axon projections were analyzed at P28-P35. Thus, only axons from neurogenic-tagged RGCs in one eye were visualized in the brain.

#### 2.2.1 Optic tract

Previous studies have shown that axons in the optic tract are chronotopically organized according to axon age and the timing of entry into the tract (Walsh et al., 1983; Walsh and Guillery, 1985). Indeed, we found that neurogenic-tagged axons occupied distinct regions of the optic tract depending on the timing of TM induction (Figure 2B). Specifically, the early tagged axons at TM11.5 were confined to the deep (furthest from the pia) and dorsal regions of the contralateral optic tract, whereas the later-tagged axons at TM15.5 were observed in the superficial and ventral regions of the contralateral optic tract. The axons labeled at TM13.5 were located in an intermediate position between the deep and superficial regions of the contralateral optic tract.

#### 2.2.2 Lateral geniculate nucleus (LGN)

The LGN is a major retinorecipient target in the thalamus (Kerschensteiner and Feller, 2024). The LGN is anatomically divided into two domains: the dorsal (dLGN) and ventral (vLGN) portions. For contralateral projections, early tagged axons at TM11.5 were observed in both the dLGN and the vLGN (Figure 2C), whereas later-tagged axons at TM13.5 or TM15.5 were confined to the dLGN. In the mouse dLGN, RGC projections are subdivided into two compartments, the medial “core” and superficial “shell” regions (Reese, 1988; Kerschensteiner and Feller, 2024). The shell receives inputs from RGCs that have direction-selective functions (Huberman et al., 2009; Kim et al., 2010; Kay et al., 2011). In our study, axons labeled at TM11.5 and TM13.5 were distributed broadly around the core region (Figure 2C). On the other hand, TM15.5 axons were preferentially found in the shell region of the dLGN.

Ipsilateral projections to the LGN were observed for TM13.5 and TM15.5, but not for TM11.5 RGCs (Figure 2C). These axons occupied the isolated medial region of the dLGN, which is considered the target region of ipsilateral projections in mice (Morin and Studholme, 2014).

#### 2.2.3 Medial terminal nucleus (MTN)

**Figure 3:**
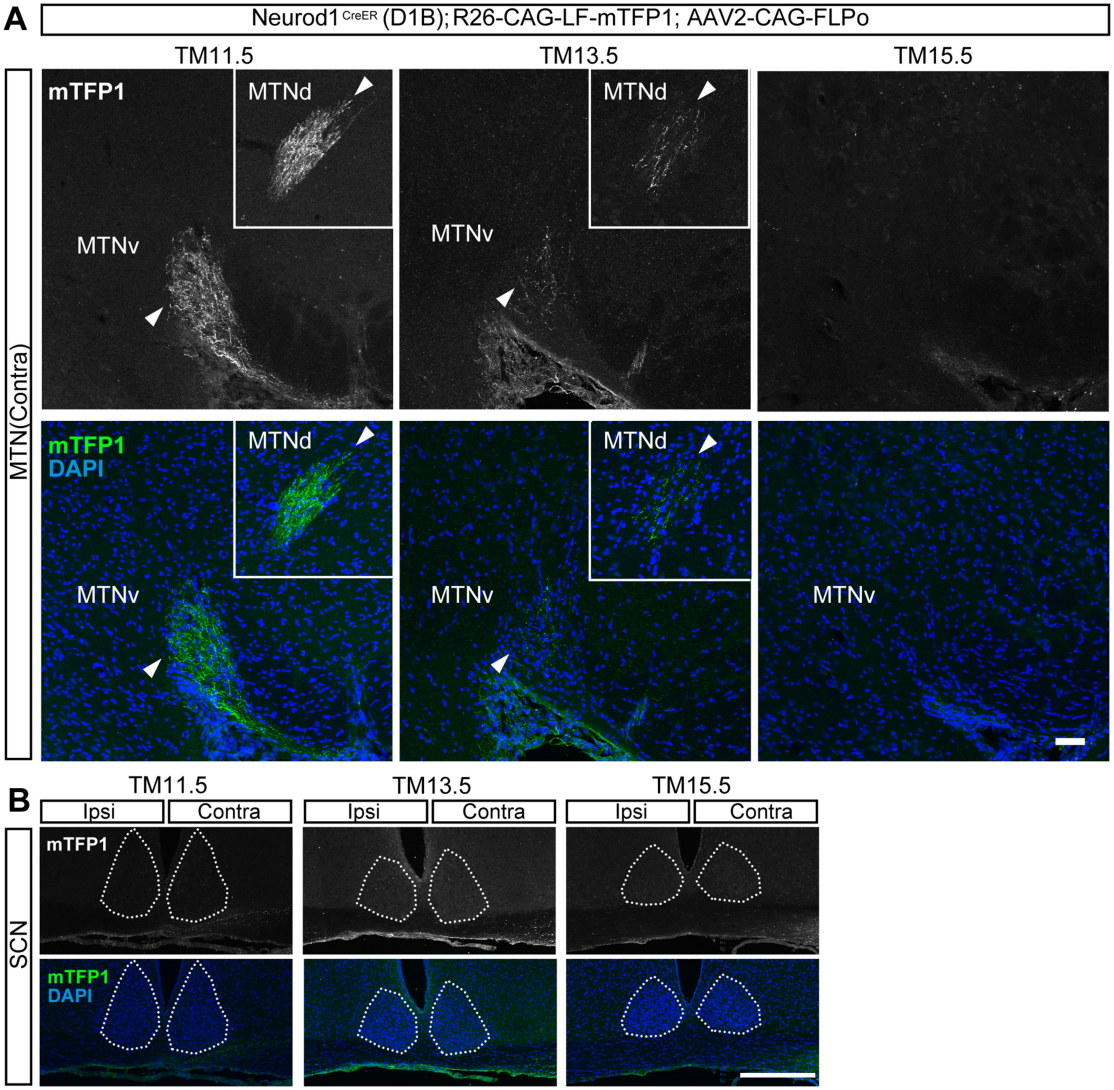
Axonal projections of the neurogenic-tagged RGCs in the MTN and SCN. **(A,B)** Labeling of TM-tagged axon arbors by mTFP1 staining (white in upper panels, green in lower panels) in brain sections using the same strategy as in Figure 2: Neurod1^CreER^ (D1B) mice were crossed with R26-CAG-LF-mTFP1 reporter mice, followed by intravitreal injection of AAV2-CAG-FLPo into one eye at the postnatal stage. TM-tagging stages are indicated at the top. DAPI staining is shown in blue. **(A)** Labeled axons in the contralateral medial terminal nucleus (MTN), indicated by arrowheads, at P29 for TM11.5, P35 for TM13.5, and P32 for TM15.5. MTNd, dorsal MTN; MTNv, ventral MTN. **(B)** Suprachiasmatic nucleus (SCN) at P28 for TM11.5, P29 for TM13.5, and P32 for TM15.5. Right, contralateral side; left, ipsilateral side. The dotted line indicates the border of the SCN. Scale bars: 50 μm **(A)** and 250 μm **(B)**.

The MTN belongs to the accessory optic system (AOS), which is responsible for proper visual acuity and velocity discrimination (Yonehara et al., 2009). Projections to the MTN were observed specifically in early tagged (TM11.5) neurons. Early tagged axons at TM11.5 projected to both the dorsal and ventral MTN (Figure 3A). Axons labeled at TM13.5 projected only sparsely to this nucleus, and no axons labeled at TM15.5 projected to the MTN.

#### 2.2.4 Suprachiasmatic nucleus (SCN)

The SCN in the hypothalamus is a target of intrinsically photosensitive RGCs (ipRGCs) and regulates circadian rhythms (Hattar et al., 2002, 2006; Hastings et al., 2018). Projections to the SCN were undetectable regardless of TM stage (Figure 3B).

#### 2.2.5 Superior colliculus (SC)

The SC in the midbrain is a major retinorecipient target that integrates visual and other sensorimotor information. Functionally distinct layers have been identified in the mouse SC (Ito and Feldheim, 2018; Kerschensteiner and Feller, 2024). The superficial layer of the SC receives direct projections from RGCs and is further subdivided into distinct sublaminas. The most superficial sublamina within this region receives projections from direction-selective RGCs, and a slightly deeper sublamina receives projections from alpha RGCs (Huberman et al., 2008; Kim et al., 2010; Hong et al., 2011; Kay et al., 2011; Martersteck et al., 2017). The deep layer of the SC, lying below the visually responsive strata, receives inputs from the brainstem and cortical areas and thus functions as a motor component of the SC (Wheatcroft et al., 2022).

**Figure 4:**
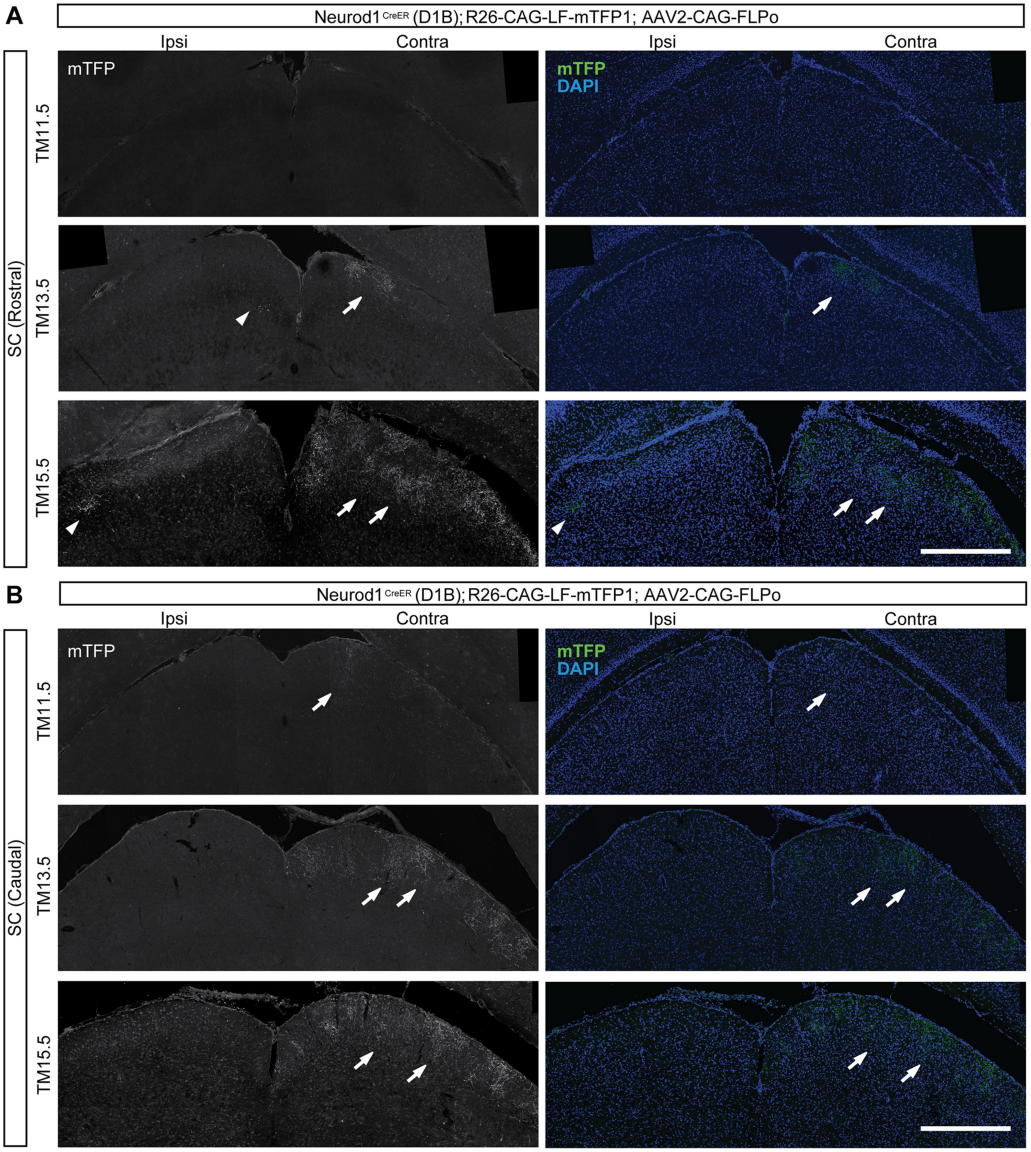
Axonal projections of the neurogenic-tagged RGCs in the SC. **(A,B)** TM-tagged axon arbors labeled by mTFP1 staining (white in left panels, green in right panels) in the superior colliculus (SC) using the same strategy as in Figure 2: Neurod1^CreER^ (D1B) mice were crossed with R26-CAG-LF-mTFP1 reporter mice, followed by intravitreal injection of AAV2-CAG-FLPo into one eye at the postnatal stage. TM-tagging stages are indicated at the top. Brain sections were analyzed at P28 for TM11.5, P35 for TM13.5, and P32 for TM15.5. DAPI staining is shown in blue. Arrowheads indicate labeled axons in the ipsilateral SC. Arrows indicate labeled axons in the contralateral SC. Right, contralateral side; left, ipsilateral side. **(A)** Rostral SC, **(B)** Caudal SC. Scale bars, 500 μm.

Contralateral projections to the SC were mostly observed at TM13.5 and TM15.5 (Figure 4A), although a few contralateral projections were also detected at TM11.5 in caudal sections (Figure 4B). The contralateral projections were confined to the superficial SC. Axons labeled at TM13.5 and TM15.5 broadly covered the superficial SC, and clear distinctions in sublaminar occupancy were not evident. Ipsilateral projections to the SC were only sparsely observed at TM13.5 and TM15.5 (Figure 4A). These axons occupied the deepest layer of the superficial SC, which corresponds to the target layer of the previously described ipsilateral RGCs in the SC (Morin and Studholme, 2014).

### 2.3 Molecular marker expression in the neurogenic-tagged RGCs

**Figure 5:**
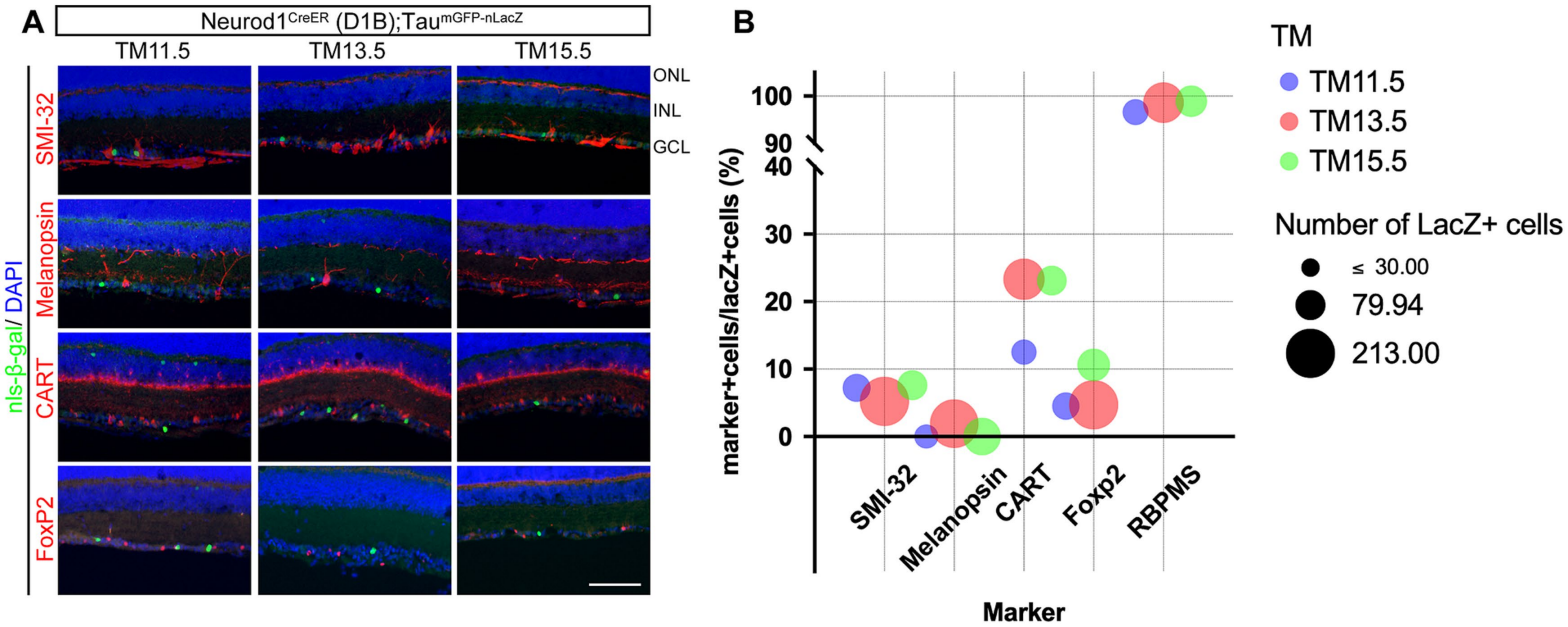
Marker expression in the neurogenic-tagged RGCs. **(A)** Immunohistochemistry for RGC subtype markers (red) combined with TM-tagged cells labeled by the nuclear-localized β-galactosidase (nls-β-gal, green) in Neurod1^CreER^ (D1B); Tau^mGFP-nLacZ^ mice at P21. TM-tagged stages are indicated at the top. DAPI staining is shown in blue. SMI-32 labels alpha RGCs; melanopsin labels ipRGCs; CART labels ON–OFF direction-selective RGCs; and FoxP2 labels FoxP2-expressing F-RGCs. ONL, outer nuclear layer; INL, inner nuclear layer; GCL, ganglion cell layer. Scale bar, 100 μm. **(B)** Percentage of double-positive cells (marker-positive and nls-β-gal/lacZ-positive) among TM-tagged cells (nls-β-gal/lacZ-positive). The TM-tagged stages are indicated by the bubble colors: TM11.5, blue; TM13.5, red; and TM15.5, green. Numbers of TM-tagged cells are indicated by the bubble size: TM11.5, 51–69 cells; TM13.5, 143–213 cells; TM15.5, 78–124 cells. Marker antibodies were the same as in **(A)**, except that RBPMS was used as the pan-RGC marker.

Molecular markers have been used to characterize RGCs and some RGC subtypes, such as alpha RGCs, ipRGCs, ON–OFF direction-selective RGCs, and F-RGCs (Hattar et al., 2002; Kwong et al., 2010; Kay et al., 2011; Rodriguez et al., 2014; Rousso et al., 2016; Krieger et al., 2017). We analyzed marker expression in the neurogenic-tagged RGCs at P21 (Figure 5). For this purpose, the neurogenic tagging driver, Neurod1^CreER^ (D1B), was crossed with the Tau^mGFP-nLacZ^ reporter line. Nuclear-localized β-galactosidase (nls-β-gal) was then detected by immunohistochemistry together with RGC markers (Figure 5A). Although the number of neurogenic-tagged RGCs (nls-β-gal-positive) varied among the TM injection stages (Supplementary Figure 1), the total number of single marker-positive RGCs remained constant. These results suggest that these molecular markers reliably identified the RGC subtypes in this analysis.

Figure 5B shows a bubble plot summarizing the results of this analysis, including the percentage and number of marker-positive cells among the neuron-tagged cells in the ganglion cell layer. Specifically, the pan-RGC marker, RBPMS, was expressed in the majority of neurogenic-tagged RGCs at all TM induction stages. The analyzed RGC subtypes accounted for less than 25% of the TM-labeled RGCs at any of the tagged time points. Among them, ON–OFF direction-selective RGCs marked by CART were slightly more abundant in TM13.5- and TM15.5-labeled RGC populations compared with those labeled at TM11.5; however, this difference did not reach statistical significance based on Fisher’s exact test (significance threshold: *p* < 0.05). The proportions of other RGC subtypes did not differ among the TM stages (Fisher’s exact test).

### 2.4 Morphological characteristics of the neurogenic-tagged RGCs

**Figure 6:**
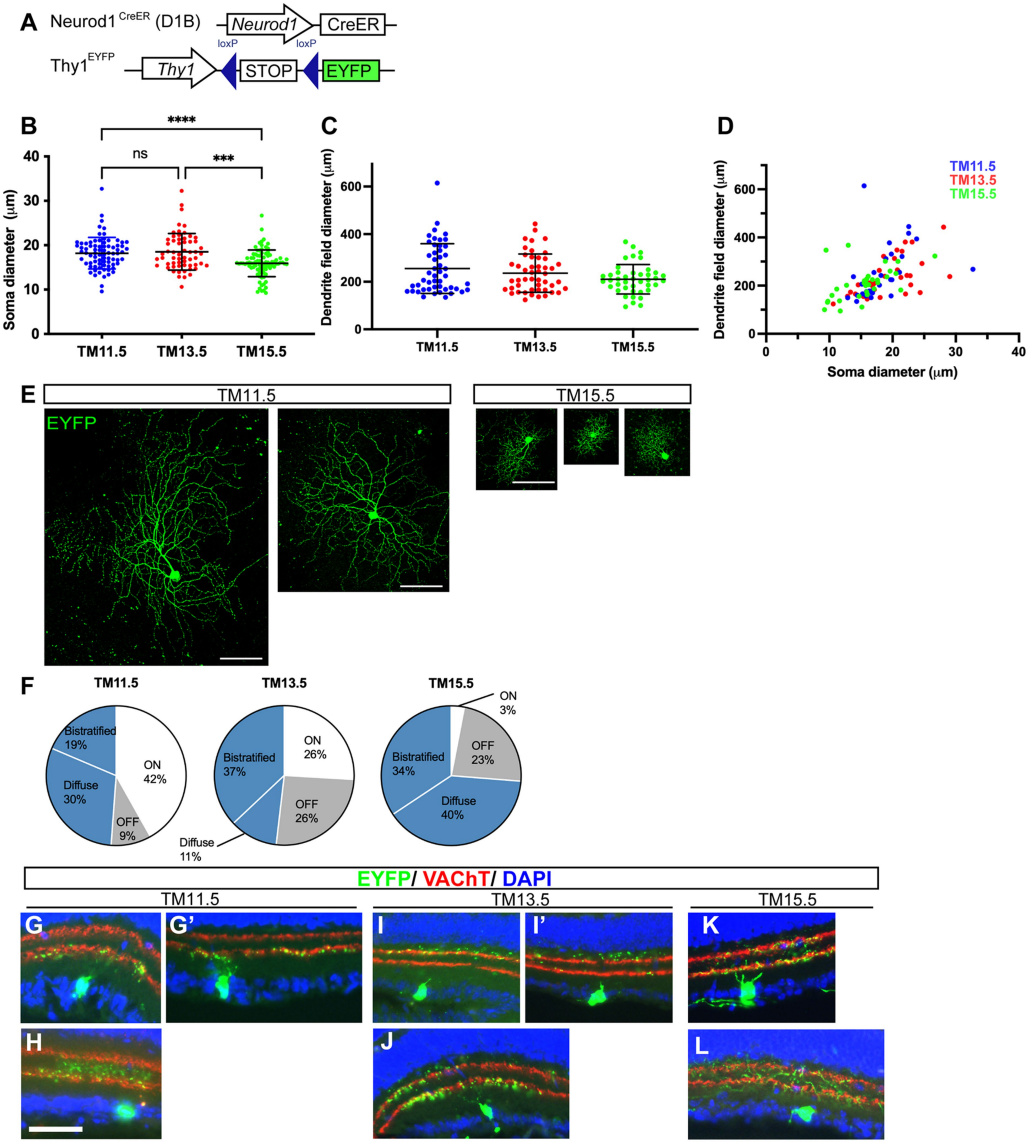
Soma size and dendrite branch lamination of the neurogenic-tagged RGCs. **(A)** Schematic gene structures of the neurogenic tagging driver Neurod1^CreER^ (D1B) and the Thy1-EYFP reporter. Tamoxifen (TM) injection was administered at E11.5, E13.5, or E15.5 to induce CreER recombination, and RGCs were analyzed at P21. **(B)** Soma diameters of neurogenic-tagged RGCs. Dots indicate individual cells; horizontal lines indicate mean ± SD. TM11.5: 18.2 ± 3.5 μm (*n* = 78 cells from 2 animals), TM13.5: 18.5 ± 4.1 μm (*n* = 59 cells from 3 animals), TM15.5: 15.9 ± 3.0 μm (*n* = 87 cells from 2 animals). ***p < 0.001; ****p < 0.0001; ns: not significant (Kruskal–Wallis with Dunn’s multiple comparisons test). **(C)** Dendritic field diameter of the neurogenic-tagged RGCs. Dots indicate individual cells; horizontal lines indicate mean ± SD. TM11.5: 255.2 ± 104.2 μm (*n* = 49 cells from 3 animals), TM13.5: 236.1 ± 80.5 μm (*n* = 48 cells from 3 animals), TM15.5: 210.3 ± 62.1 μm (*n* = 42 cells from 1 animal). **(D)** Scatter plot showing the relationship between dendritic field diameter and soma diameter. Dots indicate individual cells (TM11.5, blue; TM13.5, red; and TM15.5, green). Numbers of measured cells are as follows: TM11.5, *n* = 30 (3 eyes from 2 animals); TM13.5, *n* = 32 (4 eyes from 2 animals); TM15.5, *n* = 34 (2 eyes from 1 animal). **(E)** Representative confocal stacks of the neurogenic-tagged RGCs in flat-mounted retinas at P21. Flat-mounted retinas were immunostained with anti-GFP (green) to visualize the expression of EYFP in the soma and dendrites. TM-tagged stages are indicated at the top. Scale bars, 100 μm. **(F)** Percentages of neurogenic-tagged RGC types categorized by dendrite lamination. Numbers of cells analyzed are as follows: TM11.5, *n* = 43 cells from 5 animals; TM13.5, *n* = 54 cells from 4 animals; TM15.5, *n* = 100 cells from 3 animals. **(G–L)** Representative images of the neurogenic-tagged RGCs (green) and IPL sublaminas labeled with VAChT (red) at P21. DAPI staining is shown in blue. TM-tagged stages are indicated at the top. Scale bars, 50 μm.

We next analyzed the soma size and dendritic morphology of the neurogenic-tagged RGCs at P21 (Figure 6) because soma size and dendritic field size differ among RGC subtypes (Rockhill et al., 2002; Goetz et al., 2022). For this purpose, the neurogenic tagging driver Neurod1^CreER^ (D1B) was crossed with the Thy1-STOP-EYFP reporter line, which has been widely used to label RGCs and their dendrites in previous studies (Buffelli et al., 2003; Kim et al., 2008) (Figure 6A). The morphology of labeled RGCs was examined in flat-mount retinal preparations. We found that the soma size of TM11.5 RGCs was slightly, but significantly, larger than that of TM15.5 RGCs (Figures 6B,E). The dendritic field size showed a trend toward group differences (Figure 6C). The relationship between dendritic field diameter and soma diameter showed a positive correlation (Figure 6D; Spearman’s r: TM11.5, *r* = 0.6587, *p* < 0.0001; TM13.5, *r* = 0.5920, *p* = 0.0004; TM15.5, *r* = 0.5463, *p* = 0.0008).

The position of dendrites within retinal sublaminas is another criterion for categorizing RGC subtypes (Figures 6F–L). We examined the dendritic positioning of the neurogenic-tagged RGCs by co-immunostaining with an antibody against the vesicular acetylcholine transporter (VAChT). VAChT immunostaining labels two layers of processes from the starburst amacrine cells in the inner plexiform layer (IPL). These layers are closely associated with the ON- and OFF-type RGCs in the retina (Huberman et al., 2009; Martersteck et al., 2017). Based on the stratification of their dendrites within the IPL, we categorized RGCs into four types: (1) ON type, in which the dendritic arbor is confined to the ON sublamina (Figures 6G,G’); (2) OFF type, in which the dendritic arbor is ramified within the OFF sublamina (Figures 6I,I’); (3) bistratified type, in which dendrite branches are distinctly confined to both ON and OFF sublaminas (Figures 6J,K); and (4) diffuse types, in which the dendritic arbor is not confined to a single sublamina but spans the IPL (Figures 6H,L). The proportions of these four types were compared among TM11.5, TM13.5, and TM15.5, using a chi-square test, which showed significant differences (χ^2^ = 44.01, df = 6, *p* < 0.0001). *Post hoc* comparisons with Bonferroni correction also revealed significant differences between TM11.5 and TM13.5 (χ^2^ = 12.69, *p* = 0.0053), TM11.5 and TM15.5 (χ^2^ = 36.35, *p* < 0.0001), and TM13.5 and TM15.5 (χ^2^ = 26.18, *p* < 0.0001) (adjusted significance threshold: *α* = 0.05/3 = 0.0167). Standardized residual analysis indicated that ON-type cells were overrepresented among TM11.5 RGCs, whereas this cell type was rare among TM15.5 RGCs. In contrast, diffuse-type cells were underrepresented among the TM13.5 RGCs.

**Figure 7:**
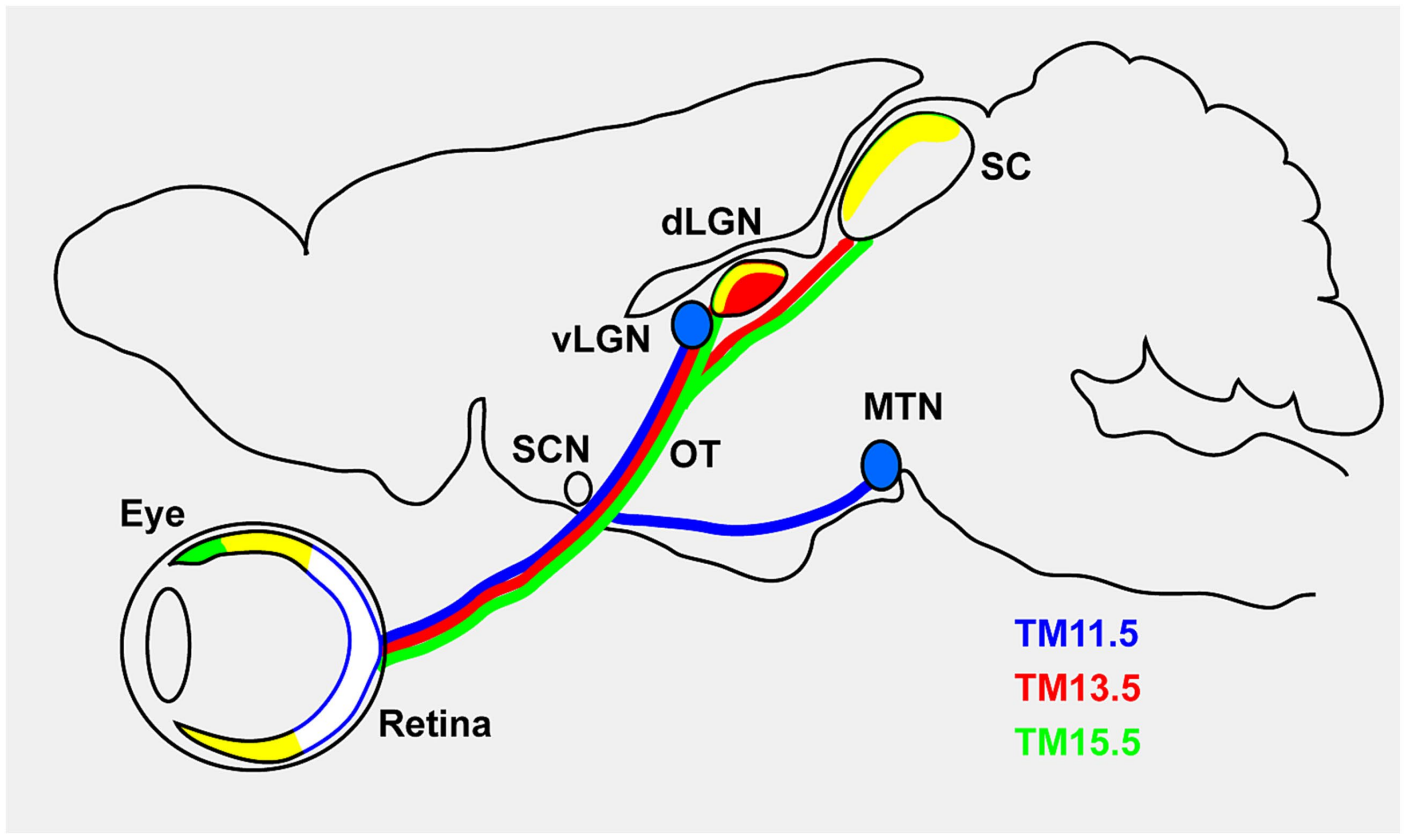
Schematic representation of axon targeting by the neurogenic-tagged RGCs. The predominant axon-targeting nuclei are shown. TM11.5 cells (blue) predominantly project to the vLGN and MTN. TM13.5 cells (red) project to the core region of the dLGN and the superficial SC. TM15.5 cells (green) predominantly project to the shell region of the dLGN and the superficial layer of the SC, but not to the vLGN or MTN. Overlapping signals are shown in additive RGB color mixing, implying that the overlap between TM13.5 (red) and TM15.5 (green) is shown in yellow, and the overlap of TM11.5 (blue), TM13.5 (red), and TM15.5 (green) is shown in white. Ipsilateral projections of TM13.5 and TM15.5 cells are not illustrated in the schematic. dLGN, dorsal lateral geniculate nucleus; vLGN, ventral lateral geniculate nucleus; SC, superior colliculus; SCN, suprachiasmatic nucleus. OT, optic tract; MTN, medial terminal nucleus.

In summary, our analysis revealed that RGC axon projections in the LGN and MTN were segregated depending on the stage of tamoxifen injection (Figure 7). In addition, morphological analyses of labeled RGCs showed that TM11.5 RGCs had slightly larger somata and included a higher proportion of ON-type RGCs compared with TM15.5 RGCs.

## 3 Discussion

Many nervous systems use differentiation timing to specify neuronal types. Previously, Osterhout et al. discussed the relationship between the differentiation timing of RGCs and their axon-targeting areas based on observations of four established RGC subtypes (Osterhout et al., 2014). The authors emphasized that RGC birth timing is related to the timing of axon arrival at the target nuclei and competition between axons, rather than RGC subtype specification. Our study expanded this idea by investigating the relationship between RGC differentiation timing and RGC subtype specification. Using the neurogenic tagging Neurod1^CreER^ (D1B) mouse line, we showed that different RGC subtypes differentiate at distinct developmental times. Our results indicate the existence of a timing-dependent differentiation mechanism for RGC specification. Here, we discuss the potential RGC subtypes characterized in our analyses (Table 1) and the potential applications of the neurogenic-tagging mouse line.

**Table 1:** Summary of potential subtypes for each neurogenic-tagged RGC and the identification method used.

TM-tagged stage	Potential subtypes	Present observations that are consistent (●) and inconsistent (□) with known subtype properties
TM11.5	ON DSGC	Axon projection to the MTN Dendrite branching (monostratified and confined to the ON sublamina) Relatively large cell body size
	ipRGC	Axon projection to the vLGN Axon occupation in the optic tract (deep and dorsal) Lack of melanopsin expression (inconsistent with ipRGC identity) Absence of projections to the SCN (inconsistent with ipRGC identity)
TM13.5	Ipsilateral RGC	Axon projection to the ipsilateral LGN and SC
	Alpha RGC	Axon projection to the core dLGN and superficial SC
	ON–OFF DSGC	Moderate dendritic field size CART expression
TM15.5	Ipsilateral RGC	Axon projection to the ipsilateral LGN and SC
	ON–OFF DSGC	Axon projection to the shell dLGN and superficial SC Dendrite branching (bistratified and confined to the ON and OFF sublaminas) CART expression

DSGC, direction-selective retinal ganglion cell; dLGN, dorsal lateral geniculate nucleus; vLGN, ventral lateral geniculate nucleus; MTN, medial terminal nucleus; SC, superior colliculus; SCN, suprachiasmatic nucleus; RGC, retinal ganglion cell; ipRGC, intrinsically photosensitive RGC.

### 3.1 Potential subtypes for TM11.5 RGCs

Axon projections to the MTN are a unique feature of ON-type direction-selective RGCs (ON DSGCs) belonging to the accessory optic system (AOS) (Yonehara et al., 2009; Dhande et al., 2013). Therefore, TM11.5 RGCs are expected to include this subtype. The AOS stabilizes retinal images by driving compensatory eye movements against head movements via projections to the MTN. Although the early generation of this subtype has not been previously described, it has been investigated as SPIG1-RGCs (Yonehara et al., 2009) (SPIG1 is also known as FSTL4), Hoxd10-RGCs (Dhande et al., 2013), and Pcdh9-Cre-labeled RGCs (Lilley et al., 2019). Our observations show that approximately 50% of TM11.5 RGCs have monostratified dendrites confined to the ON sublamina (Figure 6F). In addition, TM11.5 RGCs exhibited relatively large soma sizes. These features are consistent with the known characteristics of ON-DSGCs (Yonehara et al., 2009; Dhande et al., 2013; Lilley et al., 2019).

Another possible subtype of TM11.5 RGCs is ipRGCs. Although the ON DSGCs described above do not project to the LGN, the LGN projections in our study were clearly labeled by TM11.5 RGC axons. In particular, axon targeting of the vLGN was prominent, and these characteristics were similar to those reported for ipRGCs (Hattar et al., 2006). The occupation of axons in the deep (furthest from the pia) and dorsal regions of the contralateral optic tract also resembles the reported pattern of ipRGCs (Hattar et al., 2006). The observation that TM11.5-labeled RGCs are preferentially located in the central retina and exhibit relatively large soma sizes is consistent with the characteristics of ipRGCs. However, this possibility is not supported by our findings at the TM stage, which showed a low percentage of melanopsin-expressing RGCs and no detectable projections to the SCN.

### 3.2 Potential subtypes for TM13.5 RGCs

Because ipsilateral projections to the LGN and SC were observed from TM13.5 and TM15.5 axons, TM13.5 RGCs (as well as TM15.5 RGCs) clearly include the ipsilateral RGC population. In both the LGN and SC, axon terminals were confined to ipsilateral target regions, consistent with the findings of previous studies (Morin and Studholme, 2014). The peak of production of ipsilateral RGCs is estimated to occur around E13-E14 in mice (Marcucci et al., 2019). This time course of ipsilateral RGC production is consistent with our observations of ipsilateral projections revealed by neurogenic tagging.

There are several possible subtypes that could account for the contralateral projections of TM13.5 RGCs. Projections to the core region of the dLGN and superficial layer of the SC are consistent with alpha RGCs (Martersteck et al., 2017). Another candidate subtype is ON–OFF DSGCs, such as BD-RGCs, which are CART-positive and have moderately sized dendritic fields (Kim et al., 2010; Kay et al., 2011). As conclusive evidence is still lacking, we do not further speculate on these possibilities.

### 3.3 Potential subtypes for TM15.5 RGCs

It is highly likely that TM15.5 RGCs include ON–OFF DSGCs, such as DRD4-RGCs or W9-RGCs (Huberman et al., 2009; Kay et al., 2011). Axons of these subtypes have been shown to project to the shell region of the dLGN and superficial SC, but not to the vLGN or MTN. These RGC subtypes are CART-positive and have relatively small dendritic fields. All these characteristics are consistent with the observed properties of TM15.5 RGCs. Moreover, many TM15.5 RGCs exhibited bistratified dendrites in both the ON and OFF sublaminas of the retina, indicating that they are indeed ON–OFF RGCs. One potential discrepancy is that the birth timing of DRD4-RGCs was previously determined to be E12.5 (Osterhout et al., 2014). This timing appears slightly earlier than the stage estimated using our neurogenic tagging method.

Axonal projection analyses also suggest that TM15.5 RGCs include ipsilateral RGCs as additional subtypes. This finding is consistent with previous observations that the production of ipsilateral RGCs decreases but continues to be observed at E15 in mice (Marcucci et al., 2019).

### 3.4 Potential shortcomings and future directions

Annotation methods for RGC subtyping using immunohistochemical markers can identify only a limited subset of RGC subtypes, collectively accounting for approximately 20% of the total RGC population (Sanes and Masland, 2015; Nguyen-ba-charvet and Rebsam, 2020). In contrast, our analyses targeted many more RGC subtypes, including unidentified subtypes. Although our classification was based on a limited number of molecular markers, this approach could provide an initial framework for distinguishing these populations. The use of additional molecular and morphological markers is necessary for a more exhaustive analysis of RGC subtypes. Recent transcriptomic studies may help in further classification because they provide new combinations of numerous subtype-specific markers in RGC populations (Baden et al., 2016; Rheaume et al., 2018; Tran et al., 2019; Goetz et al., 2022). In the future, to further refine subtype identity and establish functional relevance, the neurogenic tagging mouse could be combined with electrophysiological imaging or recording, as well as with activating or silencing manipulations (Yonehara et al., 2009; Kay et al., 2011; Kofuji et al., 2016). For example, calcium imaging would enable selective labeling and functional analysis of these neurons *in vivo*. In addition, crossing the neurogenic tagging mouse line with Rosa26-loxP-STOP-loxP-DTA or DTR mice would allow selective ablation of the targeted cells. Using these mice, visual assays, such as optokinetic response tests, could reveal the functional roles of RGC subtypes *in vivo*.

Temporal precision may represent another limitation in distinguishing closely related RGC subtypes. Higher-resolution approaches, such as the direct injection of 4-hydroxytamoxifen (4-OHT), the active metabolite of tamoxifen, may allow separation within a narrower developmental window (Guenthner et al., 2013; Hatanaka et al., 2024). Unlike nucleotide-based neuronal birthdating methods, the neurogenic tagging approach used in this study does not directly provide information regarding the timing of the cell cycle exit (Llorens-Martín and Trejo, 2011; Govindan et al., 2018). This limitation may restrict certain applications but also represents an advantage in other contexts, as the two methods are based on distinct biological mechanisms.

Finally, it is possible that the Neurod1 promoter does not label all progenitors contributing to the full diversity of RGC subtypes, thereby creating bias in the current analyses of RGC subtypes. As Neurod1 has been shown to partially compensate for the loss of Math5 (Atoh7) (Mao et al., 2008), RGC differentiation is likely to involve other transcription factors.

## 4 Materials and methods

### 4.1 Animals

The Neurod1^CreER^ (D1B) mouse line (official name: *C57BL/6-Tg (Neurod1-cre/ERT2) D1BTahi*) was generated as previously described (Hirata et al., 2021). CAG-Cre mice, constitutively expressing Cre recombinase, have been described in previous studies (Sakai and Miyazaki, 1997; Sato et al., 2011). Tau-STOP-mGFP-IRES-NLS-lacZ reporter mice (RRID: IMSR_JAX: 021162) (Hippenmeyer et al., 2005), maintained on a mixed CD-1 background, were provided by Dr. Silvia Arber (Friedrich Miescher Institute for Biomedical Research) and backcrossed with C57BL/6 wild-type mice (RRID: MGI: 5295404) for at least four generations. Thy1-STOP-YFP reporter mice (*B6.Cg-Tg (Thy1-EYFP) 15Jrs/J*; RRID: IMSR_JAX:005630) (Buffelli et al., 2003) were obtained from The Jackson Laboratory. R26-CAG-LF-mTFP1 reporter mice (*B6;129S6-Gt(ROSA)26Sor^<tm1(CAG-mTFP1)Imayo>^*/ImayoRbrc; RRID: IMSR_RBRC 05146) (Imayoshi et al., 2012) were obtained from the RIKEN BRC through the National BioResource Project of the MEXT/AMED, Japan. Heterozygous Neurod1^CreER^ (D1B) mice were crossed with homozygous reporter mice. In some experiments, CAG-Cre mice were crossed with Tau-STOP-mGFP-IRES-NLS-lacZ reporter mice. Embryonic day (E) 0.5 was defined as the day a vaginal plug was detected, and the day of birth was designated as postnatal day 0 (P0). Animals of both sexes were used for the analysis. All animal care and procedures were approved by the Institutional Animal Committees of the National Institute of Genetics and conducted in accordance with the institutional guidelines.

### 4.2 Tamoxifen treatment

Tamoxifen (Sigma-Aldrich, T5648) was dissolved in corn oil (Sigma-Aldrich, C8267) at a final concentration of 9 mM together with 5 mM progesterone (Fujifilm Wako, 160-24511), and 250 μL of this solution was administered by intraperitoneal injection to staged pregnant mice. Injections were performed once on each indicated day from E11.5 to E15.5 (hereafter referred to as TM11.5 to TM15.5, respectively). Because tamoxifen treatment often delayed parturition, pups that were not delivered by E19.5 were collected by cesarean section and transferred to ICR foster mothers (RRID: MGI:5462094, Japan SLC Inc.). For dendrite morphology analyses, the injection volume of the tamoxifen solution was reduced to 100 μL at TM11.5, 50 μL at TM13.5, and 50 µL at TM15.5 per mouse to decrease the number of labeled neurons and minimize overlap.

### 4.3 Immunohistochemistry

The retina and brain for cryosections were prepared as previously described (Hirata et al., 2021). Briefly, the animals were anesthetized with isoflurane and transcardially perfused with phosphate-buffered saline (PBS) followed by 4% paraformaldehyde (PFA). The brains and eyes were dissected and further fixed with 4% PFA for 12–24 h at 4 °C. After fixation, the tissue was cryoprotected overnight in 30% sucrose in PBS and frozen in a mixture of OCT compound (Sakura Finetek) and 30% sucrose in PBS at a 2:1 ratio. Coronal sections (14 μm for retinal sections and 20 μm for brain sections) were cut using a cryostat and placed on MAS-coated glass slides (Matsunami Glass). The sections were treated with 0.5% blocking reagent (FP1020, PerkinElmer) containing 0.2–0.4% Triton X-100 (Nacalai Tesque Inc., 35501-15) for 30 min at room temperature and incubated overnight at 4 °C with primary antibodies: chicken anti- β-galactosidase (1:1000, Abcam Cat#ab9361, RRID: AB_307210), rabbit anti-GFP (1:1000, MBL International Cat#598S, RRID: AB_591816), rat anti-mTFP1 (1:500, ximbio Cat#155264), rabbit anti-RBPMS (1:100, Abcam Cat# ab194213, RRID: AB_2920590), goat anti-VAChT (1:3000, Millipore Cat#ABN100, RRID: AB_2630394), rabbit anti-Melanopsin (1:5000, Advanced Targeting Systems, Cat#AB-N38, RRID: AB_1608077), mouse anti-SMI-32 (1:3000, Covance, Cat#SMI-32P, RRID: AB_2314912), rabbit anti-FOXP2 (1:20000, Abcam, Cat#ab16046, RRID: AB_2107107), rabbit anti-CART (1:2000, Phoenix Pharmaceuticals, Cat#H-003-62, RRID: AB_2313614). The sections were then incubated for 2 h at room temperature with secondary antibodies: donkey Alexa Fluor 488-conjugated anti-rabbit IgG (1: 500–1000, Molecular Probes Cat#A-21206, RRID: AB_2535792), donkey Cy3-conjugated anti-rabbit IgG (1:1000, Jackson ImmunoResearch Labs Cat#711–165-152, RRID: AB_2307443), donkey Cy3-conjugated anti-mouse IgG (1:1000, Jackson ImmunoResearch Labs Cat#715–165-150, RRID: AB_2340813), donkey Alexa Fluor 488-conjugated anti-chicken IgY (1:1000, Jackson ImmunoResearch Labs Cat#703–545-155, RRID: AB_2340375), and donkey Cy3-conjugated anti-goat IgG (1:1000, Jackson ImmunoResearch Labs, Cat#705–165-147, RRID: AB_2307351). The sections were counterstained with DAPI (4′, 6-diamidino-2-phenylindole, Fujifilm Wako, Cat#045–30361). For staining of nucleus-localized-β-galactosidase, the sections were pre-treated for antigen retrieval by incubating in 10 mM sodium citrate with 0.05% Tween-20 (pH 6.0) at 95 °C for 20 min before blocking. For staining of flat-mounted retina, animals were fixed as described above, and a small radial slit was made at the dorsal pole of the retina for future orientation. The cornea was cut, the lens was removed, and the neural retina was detached from the eye. Dissected retinae were processed for staining, as described above.

### 4.4 Image acquisition

Fluorescent images were captured with a confocal microscope (Olympus IX81 FV1000) using FluoView software (FV-ASW version 4.02; Evident Scientific, Japan) and processed as previously described (Hirata et al., 2019). Some images (Figures 5A, 6G–L) were captured with a CCD camera (Olympus DP71) mounted on a conventional fluorescence microscope (Zeiss Axioplan 2) using the Cell Sens Standard software. Brightness and contrast were adjusted equally across the entire image using the Adobe Photoshop software.

### 4.5 EdU incorporation assay

Neurod1^CreER^ (D1B); Tau^mGFP-nLacZ^ mice that had received tamoxifen at E13.5 were used for this analysis. A thymidine analog, 5-Ethynyl-2′-deoxyuridine (EdU, Tokyo Chemical Industry Cat#E1057; 50 mg/kg body weight), was injected intraperitoneally into pregnant mice at selected time points before or after tamoxifen injection. Eyes were dissected at P0, and coronal sections (16 μm thickness) were prepared as described above. The sections were subjected to antigen retrieval and immunostaining, as previously described (Hirata et al., 2021). Primary antibodies used for immunostaining were chicken anti-β-galactosidase (Abcam Cat#ab9361, RRID: AB_307210), followed by donkey Alexa Fluor 488-conjugated anti-chicken IgY (Jackson ImmunoResearch Labs Cat#703–545-155, RRID: AB_2340375). Incorporated EdU was detected by incubation in a solution containing 0.1 M Tris (pH 7.6), 2 mM CuSO_4_, 3 μM Alexa Fluor 555 azide, triethylammonium salt (Thermo Fisher Scientific Cat# A20012), and 10 mM ascorbic acid for 40 min at room temperature.

### 4.6 Virus injections

Neurod1^CreER^ (D1B); R26-CAG-LF-mTFP1 mice were administered tamoxifen at a selected embryonic stage (TM11.5, TM13.5, or TM15.5). Juvenile mice (P8-P21) were used for the virus injection. Under anesthesia with 1.0–1.5% isoflurane, a small hole was made in the eye just below the limbus, using an insect pin to release intraocular pressure. One to two microliters of AAV2-CAG-FLPo solution (4.5 × 10^12^ genome copies/ml; Vector Biolabs, Cat# VB1313) was injected through the hole using a pulled and beveled glass pipette (Drummond Cat#5–000-1001-X10). A glass pipette was attached to a Hamilton syringe and micromanipulator to control the injection volume. Two to three weeks after virus injection, the mice were transcardially perfused with 4% PFA in PBS, and the eyes and brains were processed for immunohistochemistry as described above.

### 4.7 Quantification and statistical analysis

The number of tagged neurons containing EdU was counted using a conventional fluorescence microscope (Zeiss Axioplan 2). The number of tagged neurons expressing markers of RGC subtypes was counted from the photographed images. The proportions of cells were calculated using Microsoft Excel.

Soma and dendritic field sizes were analyzed as previously described (Berson et al., 2010; Estevez et al., 2012). Briefly, the outlines of convex polygons that minimally enclosed the somatic or dendritic profiles of individual labeled cells were traced. These outlines were exported to ImageJ software^2^ to calculate the diameter of a circle with the same area as the convex polygon. Dendritic stratification was analyzed with reference to cholinergic bands visualized using VAChT staining. For the ON and OFF subtypes, the boundary between ON and OFF was assigned at the midpoint between the two VAChT bands (approximately 50% IPL depth), as previously described (Landi et al., 2007).

Scatter and bubble plots were generated using GraphPad Prism, version 10. Statistical analyses were performed using GraphPad Prism 10 and Microsoft Excel after confirming that the data met the assumptions of the respective tests (Figures 5B, 6B–D,F). In brief, for soma diameter and dendritic field diameter measurements, Shapiro–Wilk and Levene’s tests revealed non-normal distributions and unequal variance across groups, respectively. Based on these results, the Kruskal–Wallis test with Dunn’s multiple comparison test was conducted in this analysis.

## Data Availability

The original contributions presented in the study are included in the article/Supplementary material. Further inquiries can be directed to the corresponding author.

## Footnotes

1
https://ssbd.riken.jp/neurogt/

2
http://imagej.nih.gov
