# Supplementary material for: Differentiation timing-dependent axon targeting and subtype specification in retinal ganglion cells
Source: Front Neurosci. 2026 Feb 10;20:1733811. doi: 10.3389/fnins.2026.1733811 (PMC12929473; doi:10.3389/fnins.2026.1733811)
Supplement: Supplementary file 1 [file Data_Sheet_1.pdf]

Supplementary Material

Differentiation timing-dependent axon targeting and cell subtype specification of retinal ganglion cells

Lena Iwai and Tatsumi Hirata*

*** Correspondence:** Tatsumi Hirata: tathirat@nig.ac.jp

1 Supplementary Figure

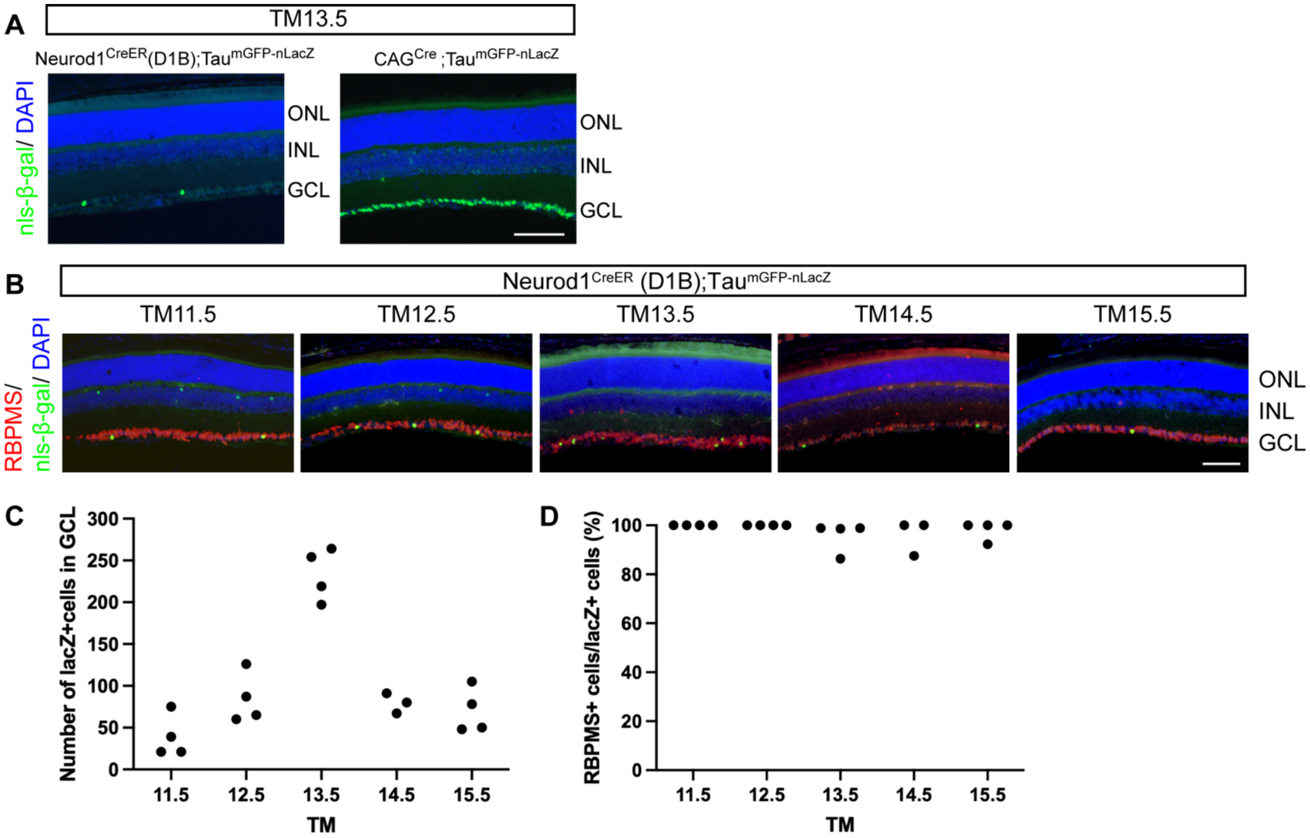

Supplementary Figure 1. Reporter expression driven by the Tau locus in this study was largely confined to RGCs.

(**A**) As observed with the Neurod1^{CreER} (D1B) driver, when a ubiquitous Cre driver (CAG-*cre*) was used with the Tau reporter line, strong reporter expression was predominantly observed in the GCL. Retinal sections at P21 were immunostained to detect reporter expression (nucleus-localized β-gal, nls-β-gal, green). TM tagging was conducted at E13.5 (TM13.5). (**B**) Reporter expression (nls-β-gal, green) was predominantly observed in the GCL and co-labeled with pan-RGC marker (RBPMS, red) in Neurod1^{CreER} (D1B); Tau$^{mGFP-nLacZ}$ mice at P21. TM-tagged stages are indicated at the top. (**A, B**) DAPI staining is shown in blue. ONL, outer nuclear layer; INL, inner nuclear layer; GCL, ganglion

cell layer. Scale bars, 100 μm. (**C**) The number of nls-β-gal-positive neurons in the GCL of Neurod1 ^{CreER} (D1B); Tau$^{mGFP\text{-}nLacZ}$ mice at P21. Retinal sections were immunostained to detect labeled cells. TM-tagged stages are indicated on the x-axis. Dots indicate values from individual mice. (**D**) The proportion of RBPMS-positive cells among nls-β-gal-positive cells in the GCL of Neurod1^{CreER} (D1B); Tau$^{mGFP\text{-}nLacZ}$ mice at P21. Retinal sections were immunostained to detect labeled cells. TM-tagged stages are indicated on the x-axis. Dots indicate values from individual mice.
